# The Effect of Osteoblast Isolation Methods from Adult Rats on Osteoclastogenesis in Co-Cultures

**DOI:** 10.3390/ijms23147875

**Published:** 2022-07-17

**Authors:** Radmila Žižková, Věra Hedvičáková, Veronika Hefka Blahnová, Věra Sovková, Michala Rampichová, Eva Filová

**Affiliations:** 1 The Czech Academy of Sciences, Institute of Experimental Medicine, Videnska 1083, 142 20 Prague, Czech Republic; vera.hedvicakova@iem.cas.cz (V.H.); veronika.blahnova@iem.cas.cz (V.H.B.); vera.sovkova@iem.cas.cz (V.S.); michala.rampichova@iem.cas.cz (M.R.); eva.filova@iem.cas.cz (E.F.); 2 Department of Chemistry, Faculty of Science, Humanities and Education, Technical University of Liberec, Studentska 1402, 461 17 Liberec, Czech Republic

**Keywords:** co-culture, osteoblast-like cell, osteoclast-like cell, mature bone, adult rat

## Abstract

Co-cultures of osteoblasts and osteoclasts are on the rise because they enable a more complex study. Diseases such as osteoporosis are related to a higher age. Thus, cell isolation from adult individuals is necessary. Osteoblasts can be isolated from the rat femur by three methods: explant culture, explant culture with enzymatic pre-treatment, or enzymatic treatment. The isolation methods yield different populations of osteoblasts which, in a co-culture with peripheral blood mononuclear cells, might result in differences in osteoclastogenesis. Therefore, we examined the differences in osteogenic markers, cell proliferation, and the metabolic activity of isolated osteoblast-like cells in a growth and differentiation medium. We then evaluated the effect of the isolated populations of osteoblast-like cells on osteoclastogenesis in a subsequent co-culture by evaluating osteoclast markers, counting formed osteoclast-like cells, and analyzing their area and number of nuclei. Co-cultures were performed in the presence or absence of osteoclastogenic growth factors, M-CSF and RANKL. It was discovered that enzymatic isolation is not feasible in adult rats, but explant culture and explant culture with enzymatic pre-treatment were both successful. Explant culture with enzymatic pre-treatment yielded cells with a higher proliferation than explant culture in a growth medium. The differentiation medium reduced differences in proliferation during the culture. Some differences in metabolic activity and ALP activity were also found between the osteoblast-like cells isolated by explant culture or by explant culture with enzymatic pre-treatment, but only on some days of cultivation. According to microscopy, the presence of exogenous growth factors supporting osteoclastogenesis in co-cultures was necessary for the formation of osteoclast-like cells. In this case, the formation of a higher number of osteoclast-like cells with a larger area was observed in the co-culture with osteoblast-like cells isolated by explant culture compared to the explant culture with enzymatic pre-treatment. Apart from this observation, no differences in osteoclast markers were noted between the co-cultures with osteoblast-like cells isolated by explant culture and the explant culture with enzymatic pre-treatment. The TRAP and CA II activity was higher in the co-cultures with exogenous growth than that in the co-cultures without exogenous growth factors on day 7, but the opposite was true on day 14. To conclude, explant culture and explant culture with enzymatic pre-treatment are both suitable methods to yield osteoblast-like cells from adult rats capable of promoting osteoclastogenesis in a direct co-culture with peripheral blood mononuclear cells. Explant culture with enzymatic pre-treatment yielded cells with a higher proliferation. The explant culture yielded osteoblast-like cells which induced the formation of a higher number of osteoclast-like cells with a larger area compared to the explant culture with enzymatic pre-treatment when cultured with exogenous M-CSF and RANKL.

## 1. Introduction

A simulation of natural conditions on in vitro models is needed to make the research on cell cultures more relevant. Therefore, monocultures are gradually replaced by co-cultures. Co-cultures of more cell types of tissue are becoming the center of interest, with an increasing desire to simulate natural conditions. Co-cultures enable the study of intercellular communication and cell signaling pathways, the influence of added drugs or supplements in a more complex way, and the study of diseases or the evaluation of materials for tissue engineering, providing more valuable insight [1,2,3,4,5,6,7]. Furthermore, more complex results from in vitro experiments would mean a reduction or even a replacement of in vivo experiments.

Bone-forming cells, osteoblasts, and bone-resorbing cells, osteoclasts, are used for co-cultures in the case of bone tissue [8]. There is still no standardized protocol for the setup of a co-culture consisting of bone cells. Published co-cultures differ in the type of used cells, the seeding procedure, the number of seeded cells, the culture media, the supplementation, and the material used for cell adhesion [1,2,3,4,5,9,10,11,12,13,14,15,16,17,18,19]. Co-cultures can be performed as a direct or indirect co-culture depending on the design [8]. In a direct co-culture, different types of cells are mixed together and cultured on the same substrate. This enables the direct contact of cells of a different type. In an indirect co-culture, two different approaches can be used. Cells can be separated by a thin membrane, allowing nutrition and signaling molecules to exchange. Alternatively, cells can be cultured in separated wells with a supernatant exchange of one for another. Indirect co-culture enables only paracrine communication between different cell types. It can be used when only paracrine communication is studied or when the analysis of separate cell types is necessary and distinguishing between them would not be possible [9,10,11,12,14,17]. However, direct cell contact is desired in most cases, as it allows for the study of cell communication and behavior because happens in vivo. The complex signaling during bone remodeling involves not only soluble but also membrane-bound molecules, for which signaling pathways are missing in indirect co-cultures [20]. Therefore, the development of bone tissue substitutes or bone in vitro models to study physiological and pathological conditions was carried out in direct co-cultures [1,2,4,7,13,15,16,18,19].

MC3T3-E1, ST2 bone forming cells were used, along with MLO-Y4 cell lines, primary osteoblasts, osteocytes, and mesenchymal stem cells (MSCs) differentiated into osteoblasts prior to the co-culture. MSCs are the most common due to their possible noninvasive collection. However, the differentiation of MSCs into osteoblasts requires time and supplementation, which can influence the subsequent co-culture. RAW264.7 bone-resorbing cells were used, along with THP-1 cell lines, macrophages isolated from the spleen or bone marrow, or mostly monocytes isolated from the peripheral blood. All the above-mentioned cells are of monocyte/macrophage lineage and have yet to be differentiated into osteoclasts. Osteoclastogenesis is induced by a macrophage colony-stimulating factor (M-CSF) and a receptor activator of nuclear factor kappa B ligand (RANKL) produced by MSCs, osteoblasts, or osteocytes, among others. Osteoclastogenesis in an in vitro co-culture can be induced by MSCs, the osteoblasts or osteocytes themselves [9,14,15,17,19], the addition of exogenous M-CSF and RANKL [2,4,10,12,13,18], or supplements inducing M-CSF and RANKL expression in bone-forming cells, e.g., prostaglandin E_2_ [3] or 1,25-dihydroxyvitamin D_3_ [4,10].

Most authors first seed bone-forming cells and then seed the precursors of bone-resorbing cells. The time interval between seeding differs from 24 h to 14 days, with 1 day used most frequently [1,2,4,9,12,13,16,18]. Only rarely was seeding opposite [3,17] or simultaneous [10,15,19]. The number of seeded cells per cm^2^ ranged from thousands to hundreds of thousands, and an important factor is also the ‘cell types’ ratio. The vast majority of papers used cell ratios in favor of bone-resorbing cells.

Primary osteoblasts are usually isolated from neonatal rats, as the isolation is easier due to low bone mineralization [21]. However, the study and treatment of diseases related to a higher age such as osteoporosis or the healing of bone substitutes in elderly individuals demand isolation from adult rats. Osteoblasts can be isolated by three different methods. Explant culture is based on enabling cell outgrowth from grinded bone pieces [22,23]. This method is easier to perform but has the disadvantage of gaining an osteoblast culture contaminated by other cell types, e.g., bone marrow cells, osteoprogenitor cells, and fibroblasts. Enzymatic isolation is more complicated, but it is a preferred method, as it gains a more homogeneous cell population [24,25,26]. The last method combines the explant culture with enzymatic pre-treatment, combining the advantages of both of these two methods [27,28].

A heterogeneous population of isolated osteoblasts, also containing bone marrow cells or fibroblasts, could be advantageous when inducing osteoclastogenesis. Mbalaviele et al. observed better osteoclastogenesis in a co-culture with undifferentiated MSCs than in the presence of osteogenic supplements supporting MSC osteogenic differentiation [13]. Additionally, the osteocyte cell line MLO-Y4 induced osteoclastogenesis better than the less differentiated osteoblast cell lines MC3T3-E1, 2T3, and OCT-1 [29]. On the other hand, Costa-Rodrigues et al. observed lower osteoclastogenesis induced by a conditioned medium from MSCs without osteogenic supplements than with osteogenic supplements. The best osteoclastogenesis was achieved by the conditioned medium from fibroblast and osteogenically induced MSCs [30]. For these reasons, we decided to examine the effect of the osteoblast isolation method on osteoclastogenesis.

Firstly, populations of rat osteoblast-like cells (rOBs) isolated by the different methods and cultured in a growth and differentiation medium were compared by means of cell proliferation, metabolic activity, osteogenic markers, and ECM mineralization. Subsequently, the effects of the isolation methods on the behavior of cells in co-cultures were evaluated. Alkaline phosphatase (ALP) activity was measured to compare rOBs in co-cultures. Osteoclastogenesis was compared by the osteoclast markers tartrate-resistant acid phosphatase (TRAP) and carbonic anhydrase II (CA II) activity measurements. The formation of rat osteoclast-like cells (rOCs) was visualized by TRAP staining. The number of formed rOCs, their area, and the number of nuclei in each rOC were analyzed using fluorescence microscopy. The differences in co-cultures were studied in the presence or absence of exogenous growth factors (GFs), M-CSF and RANKL, supporting osteoclastogenesis.

## 2. Results

### 2.1. Rat Osteoblast-Like Cells (rOBs) Isolation

The method of rOBs isolation using enzymatic treatment was inefficient on adult rat femurs. Some cells were isolated but remained rounded with poor adhesion to the cell culture flask. Only a limited number of cells adhered. However, they did not proliferate and underwent apoptosis within a few days (Figure S1). Therefore, only the cells obtained by explant culture or by explant culture with enzymatic pre-treatment were further studied.

### 2.2. rOBs Proliferation Determined by dsDNA Quantification

**Figure 1:**
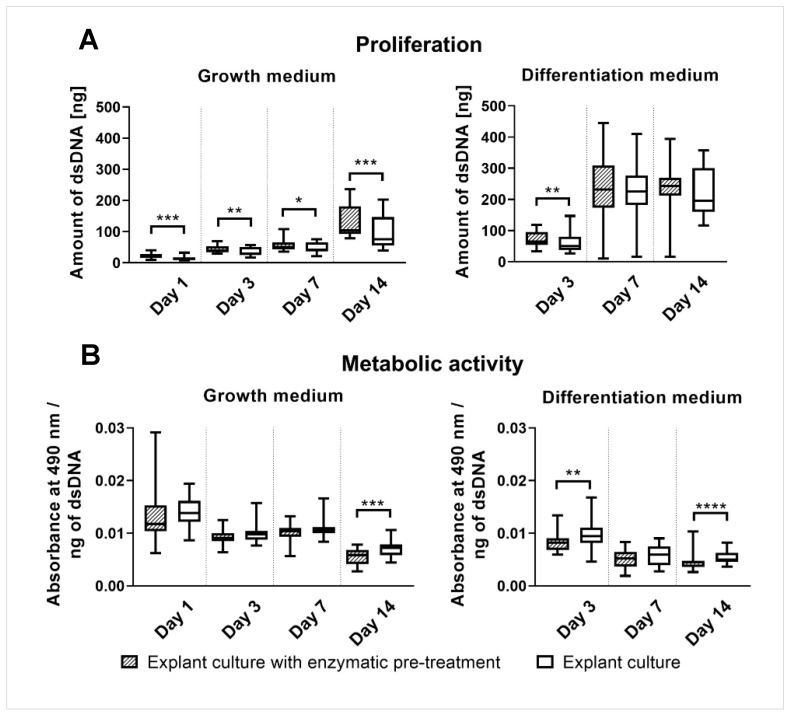
Proliferation (**A**) and metabolic activity (**B**) of rOBs isolated either by explant culture with enzymatic pre-treatment or by explant culture. Proliferation assessed by dsDNA quantification and metabolic activity measured by MTS assay and normalized to the estimated amount of rOBs were measured on days 1, 3, 7, and 14. Cells were cultured either in the growth or in the differentiation medium. The statistical difference is marked as a link above the groups. * *p* < 0.05, ** *p* < 0.01, *** *p* < 0.001, **** *p* < 0.0001.

rOBs cultured in the growth medium isolated by explant culture with enzymatic pre-treatment showed a higher amount of dsDNA than rOBs isolated by explant culture on days 1, 3, 7, and 14. rOBs cultured in the differentiation medium isolated by explant culture with enzymatic pre-treatment showed a higher amount of dsDNA than rOBs isolated by explant culture on day 3 (Figure 1A).

### 2.3. rOBs Metabolic Activity Measured by the MTS Assay

rOBs cultured in the growth medium isolated by explant culture with enzymatic pre-treatment showed lower metabolic activity than rOBs isolated by explant culture on day 14. The metabolic activity of rOBs cultured in the differentiation medium isolated by explant culture with enzymatic pre-treatment was lower than that of rOBs isolated by explant culture on days 3 and 14 (Figure 1B).

### 2.4. rOBs ALP Activity

**Figure 2:**
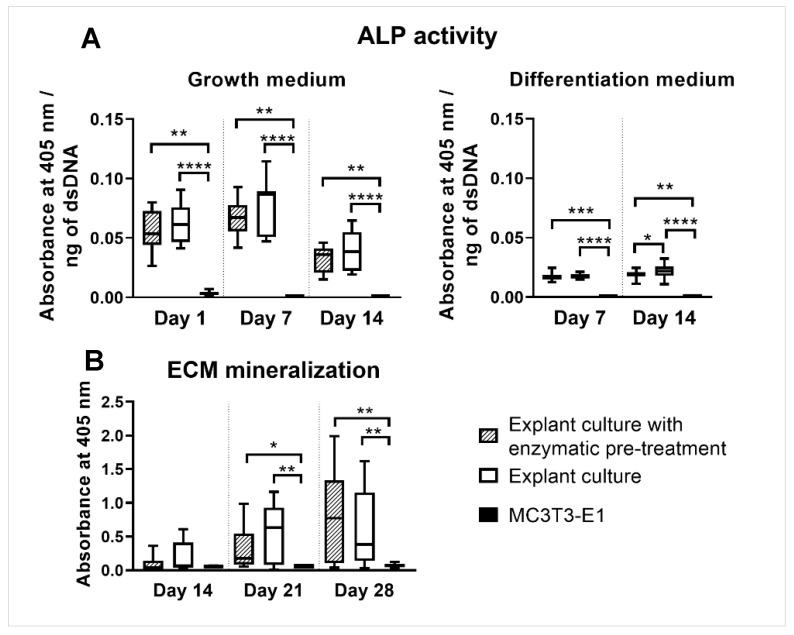
ALP activity (**A**) and quantified ECM mineralization (**B**) by rOBs isolated either by explant culture with enzymatic pre-treatment or by explant culture. MC3T3-E1 osteoblastic cell line served as a control. ALP activity normalized to the estimated amount of rOBs was assessed on days 1, 7, and 14. ECM mineralization was assessed on days 14, 21, and 28. Cells were cultured either in the growth or the differentiation medium. The statistical difference is marked as a link above the groups. * *p* < 0.05, ** *p* < 0.01, *** *p* < 0.001, **** *p* < 0.0001.

rOBs isolated by both methods cultured in a growth and differentiation medium showed comparable ALP activities on all experimental days. rOBs cultured in a differentiation medium isolated by explant culture with enzymatic pre-treatment showed lower ALP activity than rOBs isolated by explant culture on day 14. MC3T3-E1 showed the lowest ALP activity in both culture media on all experimental days (Figure 2A).

### 2.5. ECM Mineralization

The quantification of ECM mineralization promoted by rOBs isolated either by explant culture with enzymatic pre-treatment or by explant culture was comparable on all experimental days. MC3T3-E1 manifested no mineralization and thus reported the lowest measured value on days 21 and 28 (Figure 2B).

**Figure 3:**
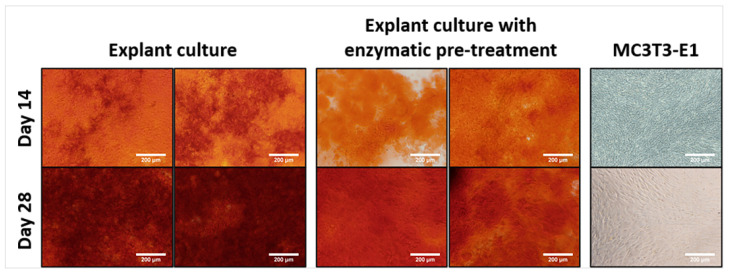
ECM mineralization stained by ARS promoted by rOBs isolated either by explant culture with enzymatic pre-treatment or by explant culture on days 14 and 28. Images from two individuals for each isolation method are shown. MC3T3-E1 served as a control. Magnification 100×, scale bar 200 μm.

ECM mineralization stained by Alizarin Red S (ARS) occurred from day 14 and became more pronounced on days 21 and 28. The control osteoblastic cell line MC3T3-E1 did not promote ECM mineralization. Images from two individuals for each isolation method are shown (Figure 3).

### 2.6. Expression of Osteogenic Markers Quantified by qPCR

**Figure 4:**
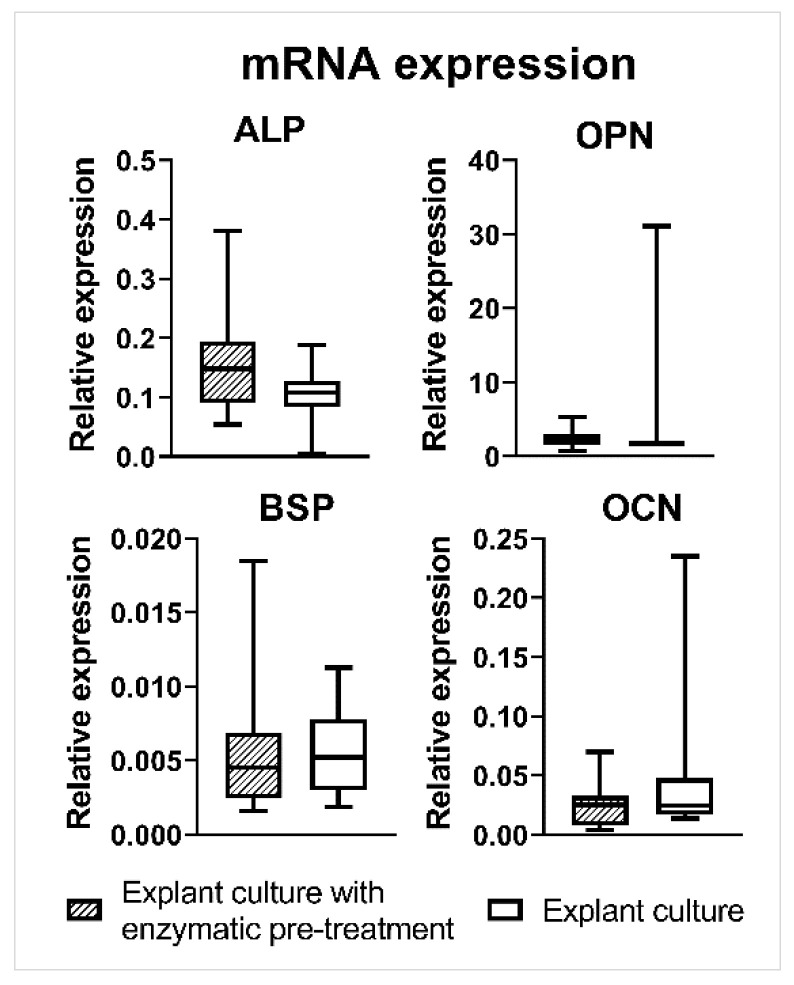
Relative mRNA expression of osteogenic markers assessed for rOBs isolated either by explant culture with enzymatic pre-treatment or by explant culture by qPCR on the day of rOBs seeding (day 0).

The mRNA expression of an early osteogenic marker, ALP, and late osteogenic markers, osteopontin (OPN), bone sialoprotein (BSP), and osteocalcin (OCN), was examined on the day of cell seeding (day 0). The expression of all osteogenic markers by rOBs isolated either by explant culture with enzymatic pre-treatment or by explant culture was comparable (Figure 4).

### 2.7. ALP Activity in Co-Cultures

**Figure 5:**
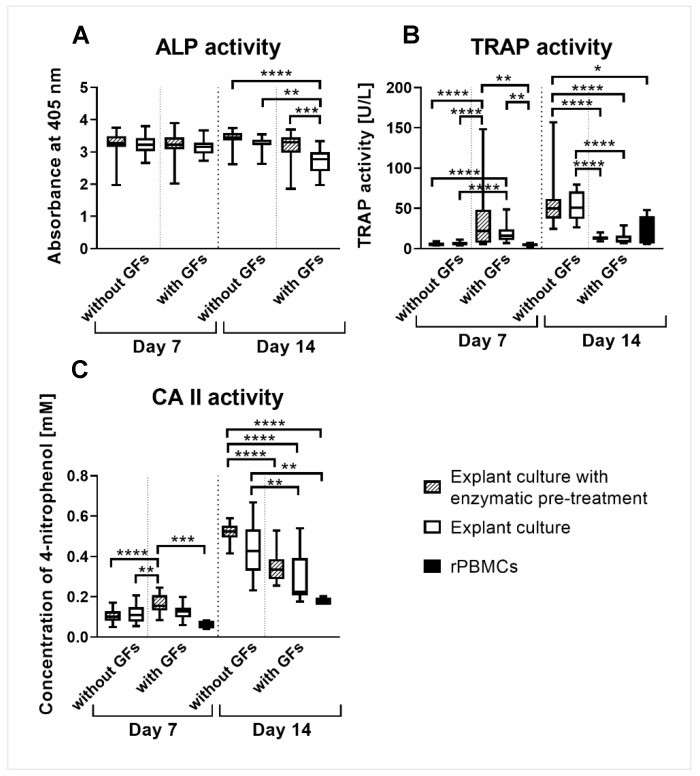
ALP (**A**), TRAP (**B**), and CA II (**C**) activity in co-cultures of rPBMCs and rOBs isolated either by explant culture with enzymatic pre-treatment or by explant culture on days 7 and 14. Cells were co-cultured with or without exogenous GFs (M-CSF, RANKL). rPBMCs monoculture served as a control of osteoclastogenesis in TRAP and CA II activity measurement and was cultured only with exogenous GFs. The statistical difference is marked as a link above the groups. * *p* < 0.05, ** *p* < 0.01, *** *p* < 0.001, **** *p* < 0.0001.

The ALP activity measured in the co-cultures of rat peripheral blood mononuclear cells (rPBMCs) and rOBs was the same on day 7, regardless of the rOBs isolation method and the presence of exogenous GFs (M-CSF, RANKL) supporting osteoclastogenesis. The co-culture of rPBMCs and rOBs isolated by explant culture cultured with exogenous GFs showed lower ALP activity than the other experimental groups on day 14 (Figure 5A).

### 2.8. TRAP Activity in Co-Cultures

The TRAP activity was higher in the co-cultures with exogenous GFs (M-CSF, RANKL) than in the co-cultures without exogenous GFs or in the rPBMCs monoculture on day 7. On day 14, the situation was the opposite; the TRAP activity was higher in the co-cultures without exogenous GFs compared to the co-cultures with exogenous GFs or the rPBMCs monoculture. There were no differences between the co-cultures with rOBs isolated either by explant culture with enzymatic pre-treatment or by explant culture (Figure 5B).

### 2.9. CA II Activity in Co-Cultures

The CA II activity was higher in the co-culture with rOBs isolated by explant culture with enzymatic pre-treatment cultured with exogenous GFs (M-CSF, RANKL) than in the co-cultures without exogenous GFs or in the rPBMCs monoculture on day 7. The co-culture with rOBs isolated by explant culture with enzymatic pre-treatment cultured without exogenous GFs had higher CA II activity than the co-cultures with exogenous GFs or the rPBMCs monoculture on day 14. Moreover, the co-culture with rOBs isolated by explant culture cultured without exogenous GFs had higher CA II activity than the co-culture with rOBs isolated by explant culture cultured with exogenous GFs or the rPBMCs monoculture (Figure 5C).

### 2.10. TRAP Staining

**Figure 6:**
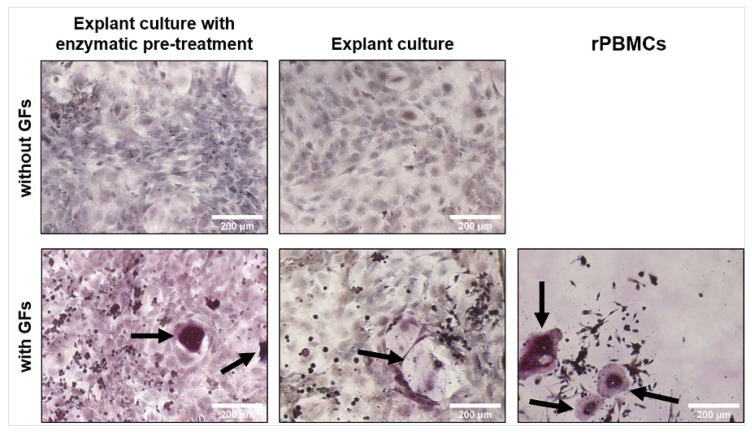
TRAP staining in co-cultures of rPBMCs and rOBs isolated either by explant culture or explant culture with enzymatic pre-treatment on day 7. Cells were cultured with or without exogenous GFs (M-CSF, RANKL). rPBMCs served as a control of osteoclastogenesis and were cultured only with exogenous GFs. TRAPs were stained by a TRAP kit and nuclei were stained by hematoxylin. Black arrows show formed rOCs. Magnification 100×, scale bar 200 μm.

**Figure 7:**
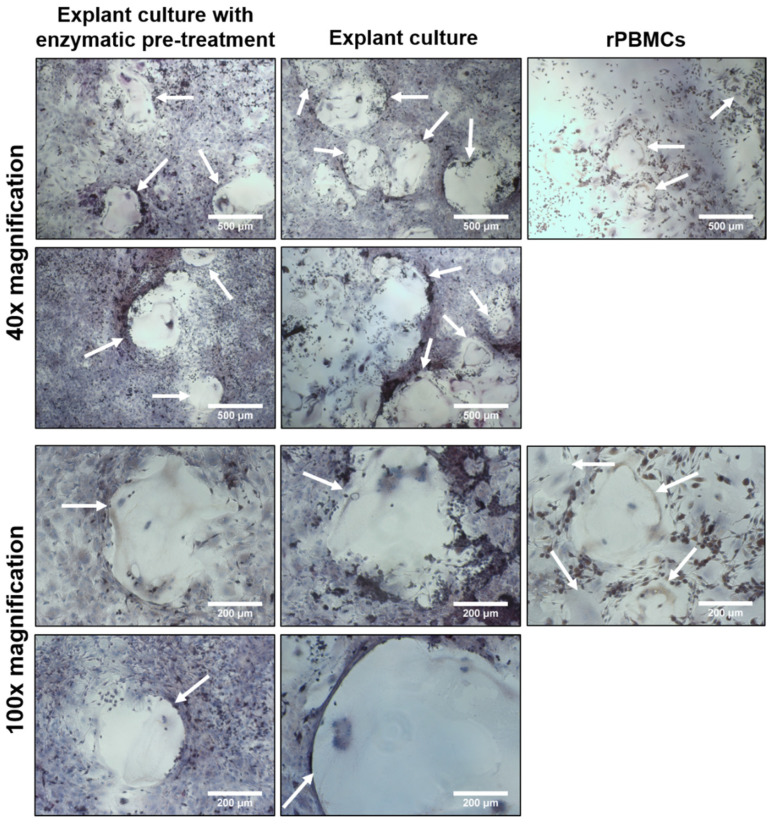
TRAP staining in co-cultures of rPBMCs and rOBs isolated either by explant culture with enzymatic pre-treatment or explant culture on day 14, cultured with exogenous GFs (M-CSF, RANKL). Images from two individuals for each isolation method are shown in a magnification of 40× and 100× (scale bar 500 μm and 200 μm, respectively). rPBMCs served as a control of osteoclastogenesis and were cultured only with exogenous GFs. TRAPs were stained by a TRAP kit and nuclei were stained by hematoxylin. White arrows show formed rOCs.

TRAP staining was conducted as an illustrative supplementary method to the above-mentioned quantitative methods. TRAP staining revealed that few rat osteoclast-like cells (rOCs) were visible on day 7 (Figure 6). Large rOCs occurred only in the co-cultures with exogenous GFs (M-CSF, RANKL) on day 14. The data from two individuals for each isolation method are shown as an example (Figure 7). In some places, apoptotic rOCs were also visible apart from large rOCs (Figure S2). The formation of rOCs was not visible in the co-cultures without exogenous GFs (Figure S3). There were no visible differences between the co-cultures with rOBs isolated by explant culture with enzymatic pre-treatment or by explant culture. rPBMCs served as a control of osteoclastogenesis (Figure 6 and Figure 7).

### 2.11. The Number of rOCs, Their Area, and the Number of Nuclei Per rOC in Co-Cultures

**Figure 8:**
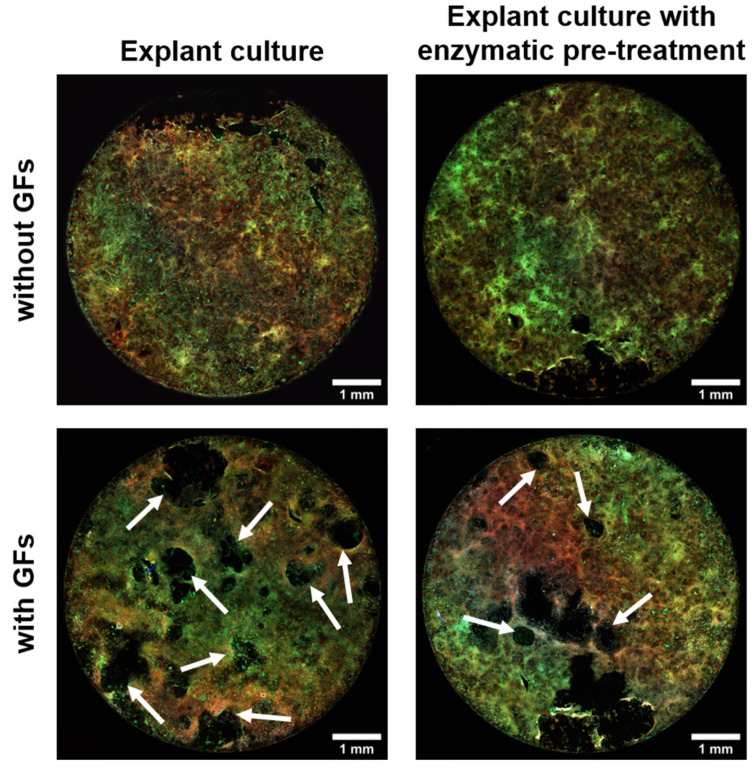
Examples of fluorescent pictures of a whole bottom of a well from which the number of formed rOCs per well, their area, and the number of nuclei per rOC were analyzed. rPBMCs and rOBs isolated either by explant culture or by explant culture with enzymatic pre-treatment were cultured with or without exogenous GFs (M-CSF, RANKL). rOCs were counted only from co-cultures with GFs on day 14. F-actin was stained by Phalloidin-iFluor555 (red), cell membranes were stained by DiOC6(3) (green), and cell nuclei were stained by DAPI (blue). White arrows show examples of the formed rOCs. Magnification 10×, scale bar 1 mm.

**Figure 9:**
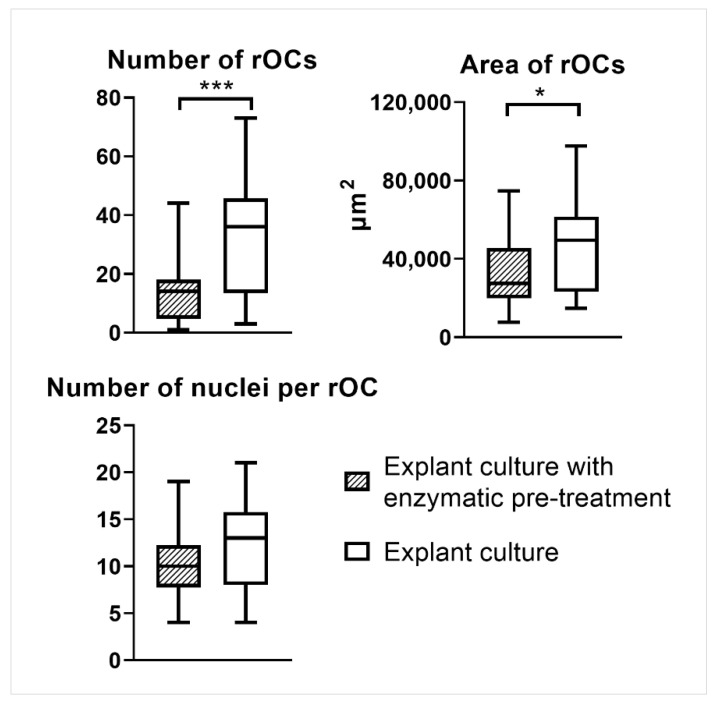
The number of formed rOCs per well, their area, and the number of nuclei per rOC in a co-culture with rOBs isolated either by explant culture with enzymatic pre-treatment or by explant culture. The shown data were analyzed for co-cultures with exogenous GFs (M-CSF, RANKL) on day 14. The statistical difference is marked as a link above the groups. * *p* < 0.05, *** *p* < 0.001.

The number of rOCs, their area, and the number of nuclei per rOC were analyzed in co-cultures with rOBs isolated either by explant culture with enzymatic pre-treatment or by explant culture using confocal microscopy (Figure 8 and Figure S4). Only the co-cultures with exogenous GFs (M-CSF, RANKL) on day 14 were analyzed as co-cultures on day 7, and the co-cultures without GFs did not form rOCs, which could be analyzed. The co-culture with rOBs isolated by explant culture formed a higher number of rOCs, and their average area was also larger. However, the numbers of nuclei per rOC were comparable, regardless of the rOBs isolation method (Figure 9).

## 3. Discussion

The known differences between the rOB populations isolated by different methods were expected to have an impact on osteoclastogenesis. This study aimed to compare three rOBs isolation methods from adult rats and evaluate their effect on the induction of osteoclastogenesis in a direct co-culture with rPBMCs. Explant culture yields a more heterogeneous cell population containing bone marrow cells and fibroblasts; therefore, we expected a different effect on osteoclastogenesis.

rOBs are mostly isolated from neonatal rats [25,26], but adult 3-month-old rats were used in this research, with the future vision to study osteoporosis and the integration of bone substitutes in adult or elderly individuals, who are known for a lower regeneration potential [31]. The isolation of rOBs by enzymatic treatment was very inefficient. The same observations were found by Robey et al., who tried to isolate cells from the adult human trabecular bone, which resulted in a limited number of isolated cells [28]. This could be due to the additional increase in bone mineralization with age [32]. The enzymatic digestion then becomes less effective. However, Gerber and ap Gwynn isolated cells from 6-day-old rats, and they also yielded a lower number of cells isolated by enzymatic isolation than by explant culture [27]. This implies that enzymatic isolation is most likely less effective than explant culture, and a higher donor age even further reduces the efficiency. Age is also connected to poor cell expansion, as the cell proliferation rate decreases with increasing age [24].

The comparison of rOBs isolated either by explant culture with enzymatic pre-treatment or by explant culture revealed some differences in proliferation and metabolic activity on some days of cultivation. rOBs cultured in a growth medium isolated by explant culture with enzymatic pre-treatment showed a higher proliferation than rOBs isolated by explant culture. In the case of the differentiation medium, the proliferation of rOBs isolated by explant culture with enzymatic pre-treatment was higher only on day 3, and then it became comparable. The proliferation of rOBs was increased by the addition of the differentiation medium from day 7. Harada et al. observed a higher proliferation of the mice osteoblastic cell line MC3T3-E1 after the addition of ascorbic acid in the same concentration we used, 50 μg mL^−1^ [33]. Takamizawa et al. also observed an increased proliferation of MG-63 human osteoblast-like cells after adding 0.25 mM ascorbic acid 2-phosphate, corresponding to 72 μg mL^−1^ [34]. The effect of ascorbic acid on primary osteoblasts was also studied. Burger et al. observed an increased osteoblast outgrowth from the bone pieces and their subsequent proliferation after the addition of 0.1 mM ascorbic acid, corresponding to 29 μg mL^−1^ [35]. However, the stimulation of proliferation by ascorbic acid is only temporary as an initial stage of subsequent differentiation [36]. Contrary to proliferation, the metabolic activity was higher for rOBs isolated by explant culture on day 14 in both the media and also on day 3 in the differentiation medium. While the proliferation of rOBs in the differentiation medium increased, the metabolic activity decreased. This indicated that the number of cells was increasing, but the contact inhibition after reaching cell confluency caused a decrease in the proliferation rate and metabolic activity per cell.

ALP activity and mineralization measurements clearly demonstrated that isolated cells are active rOBs. The ALP activity was comparable for both isolation methods, except for day 14 in the differentiation medium, where rOBs isolated by explant culture had a higher ALP activity. Jonsson et al. also observed no differences in ALP activity with human osteoblasts isolated by explant culture or explant culture with collagenase pre-treatment [24]. In our experiment, ARS staining showed strong positivity for ECM mineralization for cells isolated by both isolation methods. A higher interquartile range in the quantification of ECM mineralization on days 21 and 28 was caused by confluent cell layers, which started to peel off. As a consequence of the cell layers peeling off, larger inconsistencies appeared. No ECM mineralization and very low ALP activity were noted in the MC3T3-E1 culture. Wang et al. observed the appearance of different subclones in MC3T3-E1, which differed in terms of mineralization ability and osteogenic differentiation [37]. Additionally, Yan et al. studied the effect of an increasing passage on the osteogenic differentiation and mineralization ability of MC3T3-E1 and revealed a decreasing tendency, which completely dropped down at passage 34 [38]. The expression of the osteogenic markers ALP, OPN, BSP, and OCN was also comparable between the isolation methods. The high range of values is attributed to interindividual differences.

There were no differences in the TRAP and CA II activity measurements between the co-cultures with rOBs isolated by different methods. However, we observed higher TRAP activity in the co-cultures with exogenous GFs compared to the co-cultures without GFs on day 7, but, interestingly, the opposite was true on day 14. A similar effect was observed for CA II activity. The co-culture with rOBs isolated by explant culture with enzymatic pre-treatment cultured with exogenous GFs had higher CA II activity than the same group cultured without exogenous GFs on day 7. Nevertheless, the opposite was true on day 14. The co-cultures with rOBs isolated by explant culture had the same CA II activity on day 7 regardless of the addition of exogenous GFs, but the group cultured without exogenous GFs became higher on day 14 compared to the group with exogenous GFs. A possible explanation is that the activity of TRAP and CA II was higher on day 7 in the groups with exogenous GFs because rOC formation was observed only in those groups compared to the groups without GFs. Nevertheless, the in vitro survival of OCs is only about five days to a maximum of one week [39], although the lifespan of osteoclasts is about two to three weeks in vivo [40]. Akchurin et al. studied osteoclast formation and death in a long-term culture (15–26 days) and discovered that the number of osteoclasts oscillated during the study. The murine macrophage cell line RAW 264.7 and primary mouse bone marrow cells were treated with M-CSF and RANKL, and the increase and decrease in the number of osteoclasts were observed at an interval of five days [41]. This supports our finding of apoptotic rOCs in groups with exogenous GFs on day 14 (Figure S2). It explains the decrease in TRAP activity on day 14. However, this is in contradiction with the findings of Fuller et al. and Miyazaki et al., who reported that the addition of M-CSF and RANKL stimulated the survival of osteoclasts measured in a 24 h experiment [42,43].

A possible explanation for the increase in TRAP and CA II activity in the co-cultures without GFs on day 14 is that monocytes that do not express TRAP and CA II gradually differentiated into macrophages expressing TRAP and CA II [44,45,46]. Osteoclastogenesis in the co-cultures without exogenous GFs could be delayed in comparison to the co-cultures with exogenous GFs, and more prolonged cultivation would be necessary to confirm this hypothesis. However, layers of rOBs were already confluent on day 14 and would probably peel off during more prolonged cultivation. Cao et al. examined the effect of mice age on osteoclastogenesis. They revealed that a higher age increased osteoclastogenesis, increased the expression of M-CSF and RANKL, and decreased the expression of osteoprotegerin by osteoblasts [18]. This implies that an increase in the age of used rats would result in unnecessary M-CSF and RANKL supplementation, inducing osteoclastogenesis in 14 days.

The TRAP activity was higher in the co-cultures with exogenous GFs than in the control rPBMC monoculture on day 7. The same observation was found for CA II activity but only for the co-culture with rOBs isolated by explant culture with enzymatic pre-treatment. We assume that the presence of rOBs supported the differentiation of rPBMCs better than the addition of only exogenous GFs, as in the case of the rPBMC monoculture. The short survival of formed rOCs in vitro in the rPBMCs group, as discussed above, most likely resulted in the same TRAP and CA II activity as that in the groups with exogenous GFs and a lower activity compared to that of the groups without exogenous GFs on day 14.

TRAP staining did not reveal substantial differences between the isolation methods. However, no rOCs were observed in the groups without exogenous GFs, implying that the rOBs production of M-CSF and RANKL was insufficient to form visible rOCs. Despite no visible rOCs, the differentiation of rPBMCs and the formation of pre-osteoclasts cannot be excluded. Pre-osteoclasts cannot be distinguished morphologically, and the expression of TRAP and CA II enzymes on day 14 indicates the formation of cells capable of enzyme expression.

The only difference in the osteoclastogenesis in the co-cultures differing in the rOBs isolation method was in the number and area of formed rOCs when cultured with exogenous GFs. The co-culture with rOBs isolated by explant culture manifested a higher number of rOCs, with a larger area than the co-culture with rOBs isolated by explant culture with enzymatic pre-treatment. The effect could be caused by the more heterogeneous population of rOBs isolated by explant culture. The presence of MSCs and fibroblasts could improve osteoclastogenesis [13,29,30]. The comparable TRAP and CA II activities between these two groups could be the result of the differentiation of monocytes to macrophages, which are not counted into rOC cells but express TRAP and CA II.

Our findings suggest that the explant culture and explant culture with enzymatic pre-treatment are both suitable isolation methods to obtain rOBs from the femurs of adult 3-month-old rats. The explant culture with enzymatic pre-treatment yielded cells with a higher proliferation. rOBs obtained by both isolation methods were capable of the induction of osteoclastogenesis in a co-culture with rPBMC after stimulation with exogenous GFs, M-CSF, and RANKL. Formed rOCs were not visible without the addition of exogenous GFs, but cells capable of TRAP and CA II expression were present in the co-culture on day 14. rOBs isolated by explant culture cultured with exogenous GFs supported the formation of a higher number of rOCs with a larger area than the rOBs isolated by explant culture with enzymatic pre-treatment, but the measured activities of TRAP and CA II enzymes were comparable.

## 4. Materials and Methods

### 4.1. Cell Culture Conditions

Culture flasks were treated with a Collagen Coating Solution (Cat. No. 125-50, Sigma-Aldrich, St. Louis, MO, USA), according to the manufacturer’s instructions, in order to improve the initial rOBs adhesion. Two types of culture media were used for the culture of rOBs and the mice osteoblastic cell line (MC3T3-E1; Sigma-Aldrich, St. Louis, MO, USA), growth medium, and differentiation medium. The growth medium for rOBs was Dulbecco’s Modified Eagle’s Medium (DMEM; Cat. No. D6429, Sigma-Aldrich, St. Louis, MO, USA), supplemented with 10% fetal bovine serum (FBS; Cat. No. 10270106, Gibco, Waltham, MA, USA), 100 U mL^−1^ penicillin, and 100 μg mL^−1^ streptomycin (P/S; Cat. No. P4333, Gibco, Waltham, MA, USA). The growth medium used for the culture of MC3T3-E1 (Cat. No. 99072810, Sigma-Aldrich, St. Louis, MO, USA) was Minimum Essential Medium—Alpha Eagle (αMEM; Cat. No. BE02-002F, Lonza, Switzerland), supplemented with 10% FBS and P/S. The differentiation medium for rOBs or MC3T3-E1 consisted of a growth medium supplemented with 2 mM β-glycerophosphate (Cat. No. 50020, Sigma-Aldrich, St. Louis, MO, USA), 50 μg mL^−1^ L-ascorbic acid 2-phosphate (Cat. No. A8960, Sigma-Aldrich, St. Louis, MO, USA), and 10 nM dexamethasone (Dexamed 4 mg mL^−1^). rPBMCs were cultured in the growth medium DMEM without L-glutamine (Cat. No. ECB7501L, EuroClone, Via Figino, Italy), supplemented with 2 mM L-glutamine (Cat. No. 250300024, Gibco, Waltham, MA, USA), 10% FBS, and P/S. Co-cultures of rOBs and rPBMCs were performed in a mixture of the growth media DMEM (Sigma-Aldrich) supplemented with 10% FBS and P/S and DMEM without L-glutamine (EuroClone) supplemented with 2 mM L-glutamine and 10% FBS and P/S in a ratio of 1:1. Co-cultures were performed with or without the addition of exogenous GFs 25 ng mL^−1^ M-CSF (Cat. No. 300-25, PeproTech, London, UK) and 30 ng mL^−1^ RANKL (Cat. No. 310-01, PeproTech, London, UK). rPBMCs were only cultured with M-CSF and RANKL GFs. All the cells were cultured at 37 °C and under a 5% CO_2_ atmosphere and a high humidity.

### 4.2. rOB Isolation Methods

**Figure 10:**
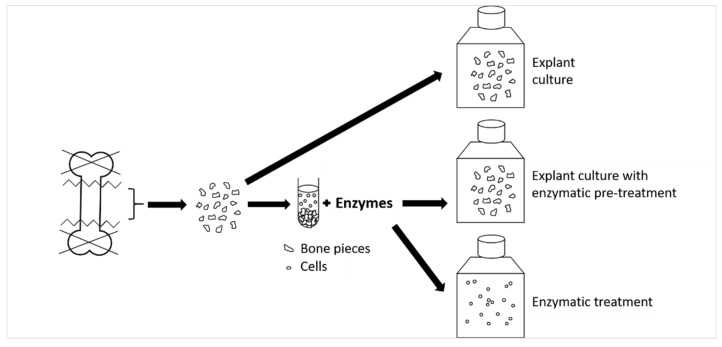
Schematic representation of the used rOBs isolation methods: explant culture, explant culture with enzymatic pre-treatment, and enzymatic treatment.

rOBs were isolated from both femurs of 3-month-old Wistar female rats by different methods: explant culture, explant culture with enzymatic pre-treatment, and enzymatic treatment. Six individuals were used for each method. The Ethical Principles and Guidelines for Scientific Experiments on Animals were respected throughout the study. The maintenance and handling of the experimental animals followed the EU Council Directive (86/609 EEC), and the animals were treated in accordance with the principles of the Care and Use of Animals and Czech Animal Protection Law No. 246/92. The femurs were collected and processed in the cooled growth medium DMEM (Sigma-Aldrich, USA) with P/S. The femurs were cleared off from the periosteum and soft tissue residues by a scalpel. The epiphyses were cut off, and the bone marrow was flushed out by DMEM with P/S. The femurs were cut into fragments of approximately 2 × 2 mm or smaller. The fragments were vortexed three times in DMEM with P/S to remove residual bone marrow and soft tissue. The next step followed one of the three methods below. For schematic representation, see Figure 10.

Explant culture

The bone fragments from two femurs of one individual were transferred to a 75 cm^2^ culture flask with 15 mL of rOBs growth medium and left still for 2 days. After outgrowing the cells, the flask was shaken to redistribute the bone fragments to cover any remaining empty spaces. This approach enabled more cells to outgrow while preventing any overgrowing of the cell islets formed around the bone fragments.

Explant culture with enzymatic pre-treatment

The bone fragments from the two femurs were enzymatically digested by 1 mL of 5× trypsin-EDTA solution (Cat. No. 59418C, SAFC Biosciences, Saint Louis, MO, USA) for 10 min in 37 °C while shaking. The solution was discarded, and the bone fragments were rinsed with DMEM with P/S. Secondly, the bone fragments were digested in 1 mL of 300 U mL^−1^ of collagenase type I (Cat. No. 17018029, Gibco, USA) solution in DMEM for 30 min at 37 °C while shaking. The solution was discarded, and the bone fragments were rinsed with DMEM with P/S. For the third stage, 1 mL of collagenase type I solution was added for 60 min at 37 °C while shaking. The bone fragments were then rinsed with DMEM with P/S and transferred to a 75 cm^2^ culture flask with 15 mL of growth medium. The flask was treated in the same manner as the flask with explant culture.

Enzymatic treatment

The bone fragments were digested by the above-mentioned procedure. Following the last digestion, the solution containing the cells was centrifuged at 300 *g* for 5 min at room temperature (RT), and the pellet was transferred to a 75 cm^2^ culture flask with 15 mL of growth medium.

The isolated cells in the culture flasks were observed by light microscopy using the microscope Olympus IX51 with a digital camera DP80.

### 4.3. Rat Peripheral Blood Mononuclear Cells (rPBMCs) Isolation

**Figure 11:**
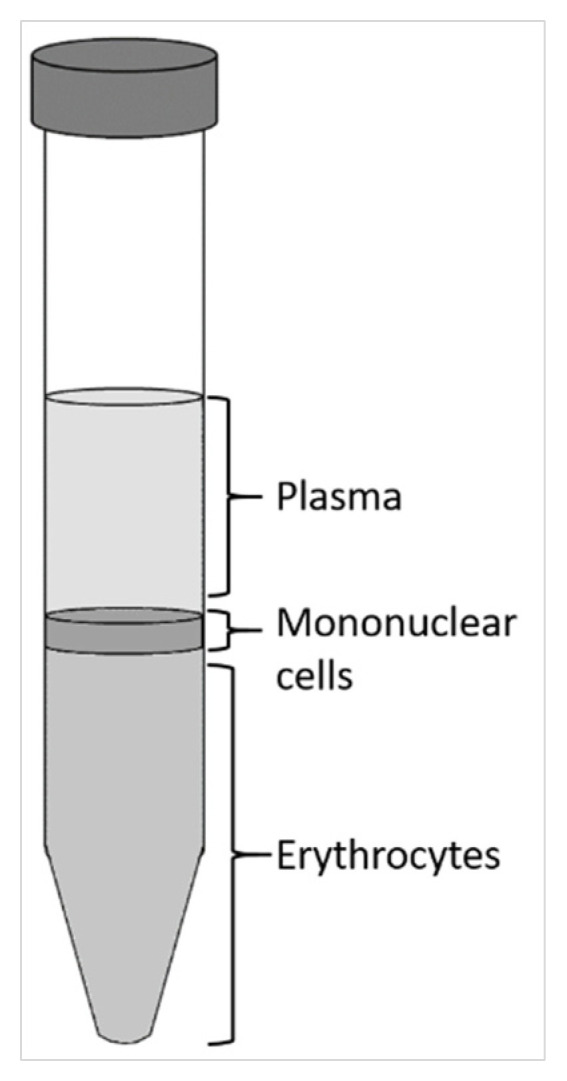
Schematic representation of layers separated after density gradient centrifugation. The lower layer contains erythrocytes, the middle layer contains mononuclear cells, which were collected, and the upper layer contains plasma.

rPBMCs were isolated from the whole blood of 3-month-old Wistar female rats by Ficoll density gradient centrifugation. Blood was obtained during isoflurane anesthesia by cardiac puncture blood collection into a syringe with 500 IU of heparin. The blood was mixed with Hanks’ Balanced Salt solution (HBSS, Gibco, USA) in a ratio of 1:1 and layered over Ficoll–Paque (Sigma-Aldrich, USA) in ratio of 4:3 without mixing. After 30 min of density gradient centrifugation at 360 g, a layer containing rPBMCs was collected (Figure 11) and rinsed three times with HBSS. Each rinse was followed by centrifugation at 16 °C for 10 min at 408 *g*, 283 *g*, and 116 *g*, respectively. After the final rinse, the pellet was resuspended in rPBMCs growth medium, and the cells were frozen for later use.

### 4.4. rOBs Seeding for Phenotypic Evaluation

First, 1.5 × 10^4^ cells cm^−2^ of the second passage of rOBs or MC3T3-E1 were seeded in 250 μL of a growth medium specific for each cell type in a 96-well plate. MC3T3-E1 were used as a control cell type. The cells were cultured in either a growth or differentiation medium. In the culture with a differentiation medium, the cells were initially cultured in a growth medium; a differentiation medium was then added after reaching cell confluency, which was 2 days after cell seeding. Both media were changed twice per week.

### 4.5. Cell Seeding for Co-Cultures

rOBs of the second passage were seeded in a density of 5.9 × 10^3^ cells cm^−2^ in 125 μL of rOBs growth medium in a 96-well plate. The next day, rPBMCs of passage 0 were added to the culture in a density of 5.9 × 10^4^ cells cm^−2^ in 125 μL of rPBMCs growth medium. The control monoculture of rOBs was seeded in a density of 5.9 × 10^3^ cells cm^−2^ in 250 μL of rOBs growth medium. The control monoculture of rPBMCs was seeded in a density of 2.9 × 10^5^ cells cm^−2^ in 250 μL of rPBMCs growth medium. rPBMCs and co-cultures performed in the presence of exogenous GFs were seeded with added M-CSF. One day after rPBMCs seeding, the growth medium was changed in all the groups to remove non-adhered cells, and RANKL was added where applicable. The day of rPBMCs seeding was marked as day 0. The cells were co-cultured for 14 days. Half the volume of the growth medium was changed twice a week.

### 4.6. rOBs Proliferation Determined by dsDNA Quantification

The proliferation of rOBs isolated by different methods was analyzed 1, 3, 7, and 14 days after seeding by quantification of the dsDNA by the Quant-iT^TM^ dsDNA Assay Kit at high sensitivity (Invitrogen, Waltham, MA, USA). The cells were lysed in 100 μL of lysis buffer (0.2% *v*/*v* Triton X-100, 10 mM Tris (pH 7.0), and 1 mM EDTA) per well. The cell lysates underwent three freeze-thaw-shaking cycles. A total of 20 μL of the cell lysate was analyzed. A total of 200 μL of the reaction buffer with a fluorescence probe was added to the samples, and fluorescence was measured at ex./em. 485/523 nm using the Tecan Infinite M200 Pro multiplate fluorescence reader. The amount of dsDNA present in the samples was calculated from a calibration curve.

### 4.7. rOBs Metabolic Activity Measured by the MTS Assay

The metabolic activity of rOBs isolated by different methods was assessed on days 1, 3, 7, and 14. The medium was removed and replaced by 100 μL of fresh growth medium and 20 μL of MTS solution, prepared according to the manufacturer’s instructions (CellTiter 96^®^ Aqueous MTS Reagent Powder, Promega corp., Madison, WI, USA), per well. The cells were incubated in a CO_2_ incubator with 5% CO_2_ at 37 °C for 2 h. After incubation, 100 μL of the solution was transferred to a clear well of a 96-well plate. The absorbance was measured at 490 nm, with the reference wavelength at 690 nm, using the Tecan Infinite M200 Pro microplate reader. The reference wavelength absorbance, together with the absorbance of the growth medium without cells, was subtracted from the measured absorbance. The resulting values were normalized to the amount of dsDNA in a corresponding well.

### 4.8. Alkaline Phosphatase (ALP) Activity

The activity of ALP was analyzed on days 1, 7, and 14 using a 1-Step^TM^ PNPP Substrate Solution kit (Thermo Fisher Scientific, Waltham, MA, USA). The medium was removed, and 100 μL of the commercial solution was added. The cells were incubated for 30 min in the dark in RT. The reaction was stopped by the addition of 50 μL of 2N NaOH. Then, 100 μL was transferred to a clear 96-well plate, and the absorbance was measured at 405 nm using the Tecan Infinite M200 Pro microplate reader. The absorbance of the Substrate Solution was subtracted from the measured absorbance. In the case of rOBs characterization, the resulting values were normalized to the average amount of dsDNA in a corresponding sample group.

### 4.9. ECM Mineralization

Mineralization of the ECM by rOBs was visualized by Alizarin Red S staining (ARS), which forms a red complex with deposited calcium. The ARS staining was performed in a differentiation medium only on days 14, 21, and 28. The cells were rinsed with phosphate buffered saline (PBS) and fixed with 10% *v*/*v* formaldehyde for 15 min. After fixation, the cells were twice rinsed with deionized water (dH_2_O) and stained with a 40 mM solution of ARS of pH 4.1–4.3. After 30 min of incubation in RT, the cells were then rinsed with dH_2_O and visualized under the light microscope Olympus IX51 with a digital camera DP80. The stained samples were then dissolved in cetylpyridinium chloride (35 mg mL^−1^, pH 7.4–7.8) while shaking at 37 °C for 2 h. A total of 100 μL was transferred to a clear well, and the absorbance was measured at 405 nm using the Tecan Infinite M200 Pro microplate reader. The absorbance of the cetylpyridinium chloride solution was subtracted from the measured absorbance.

### 4.10. The Expression of Osteogenic Markers Quantified by qPCR

The expression of osteogenic markers was evaluated using quantitative polymerase chain reaction (qPCR). RNA was isolated from 1 × 10^5^ cells separated from the cell suspension during seeding (day 0) using an RNeasy Mini kit (Qiagen GmbH, Hilden, Germany), according to the manufacturer’s instructions. The isolated mRNA was reverse transcribed to cDNA using a RevertAid H Minus First Strand cDNA Synthesis Kit (Thermo Fisher Scientific, Waltham, MA, USA), following the manufacturer’s protocol. The samples were kept on ice during the procedure. Subsequently, the expression of ALP (Rn01516028_m1), OPN (Rn00593931_m1), BSP (Rn01450118_m1), and OCN (Rn00566386_g1; all TaqMan, Thermo Fisher Scientific, Waltham, MA, USA) was measured. The TaqMan Gene Expression Master Mix (Thermo Fisher Scientific, Waltham, MA, USA) and qPCR Grade Water (Thermo Fisher Scientific, Waltham, MA, USA) was added to each sample. Light Cycler 480 (Roche, Basel, Switzerland) was used to measure the fluorescence intensity. The parameters of qPCR were set as follows: activation—95 °C for 10 min; amplification—95 °C for 10 s, 60 °C for 10 s (50 cycles); termination—40 °C for 1 min. As the relative quantification 2^−ΔCp^ method was used, the values were normalized by the 14-3-3 protein zeta/delta (YWHAZ; Rn00755072_m1) housekeeping gene. In between the isolation of RNA, cDNA synthesis, and qPCR, the samples were stored in a freezer at −80 °C.

### 4.11. TRAP Staining

The presence of TRAP as a marker specific for rOCs was visualized by Acid Phosphatase and the Leukocyte (TRAP) kit (Sigma-Aldrich, St. Louis, MO, USA) on days 7 and 14. Cell fixation and staining were performed according to the manufacturer’s instructions. Briefly, the cells were fixed with a fixative solution (citrate solution, acetone, formaldehyde) for 30 s and stained with a staining solution (naphthol AS-BI phosphoric acid, fast garnet GBC base solution, acetate buffer, tartrate buffer, sodium nitrite) for 1 h at 37 °C. Nuclei were stained with a hematoxylin solution, Gill No. 3, for 2 min and rinsed with tap water. Microscope Olympus IX51 with a digital camera DP80 was used for visualization.

### 4.12. TRAP Activity Assay

The TRAP activity was measured in the cell lysates on days 7 and 14. The cells were lysed in 100 µL of 1% Triton X-100 in PBS and left on ice for 50 min. The lysate was frozen and stored for later analysis. After thawing, the lysate was shaken for 10 min, and the well bottom was scraped by a tip. A total of 10 µL of the cell lysate was transferred to a clear well, and 50 µL of the reaction buffer (2.5 mM naphthol AS-BI phosphate, 100 mM sodium acetate, 50 mM sodium tartrate dihydrate; pH = 6.1) was added. The samples were incubated at 37 °C for 30 min. A total of 150 µL of 0.1 M NaOH was added to stop the reaction. Fluorescence was measured at ex./em. 405/520 nm using the multiplate fluorescence reader Tecan Infinite M200 Pro. TRAP activity was calculated from a calibration curve of standards from the Bone TRAP (TRAcP 5b) ELISA Immunoassay kit (IDS, UK).

### 4.13. CA II Activity Assay

The presence of the enzyme CA II, a marker of rOCs, was detected by its activity in converting 4-nitrophenyl acetate to 4-nitrophenol on days 7 and 14. The analyzed cell lysate was the same as the one used in the TRAP activity assay. A total of 50 µL of the cell lysate was mixed with 50 µL of the reaction buffer containing the substrate (12.5 mM TRIS, 75 mM NaCl, 2 mM 4-nitrophenyl acetate; pH = 7.5) and incubated for 1 h in RT. The concentration of the produced 4-nitrophenol was indirectly measured by absorbance at 405 nm, using the microplate reader Tecan Infinite M200, and calculated from the 4-nitrophenol (0.1–0.5 mM) calibration curve.

### 4.14. Confocal Microscopy—Analysis of the Number of rOCs, Their Area, and the Number of Nuclei

Confocal microscopy was used to capture the whole bottom of a well in order to count the number of formed rOCs, the area of each rOC, and the number of nuclei of each rOC in ImageJ (NIH, USA). The cells were fixed with 2% paraformaldehyde in PBS for 10 min and rinsed with PBS. Permeabilization was done with 0.1% Triton X-100 in PBS for 15 min and with 1% Tween 20 in PBS for 15 min. The cells were rinsed with PBS and stained with 0.1 μg mL^−1^ of DAPI (Sigma-Aldrich, St. Louis, MO, USA) staining nuclei, 10 μg mL^−1^ of DiOC6(3) (Invitrogen, Waltham, MA, USA) binding to cell membranes, and 1× Phalloidin-iFluor 555 (ab176756; 1:1000 dilution, Abcam, Cambridge, UK) binding to filamentous actin at RT for 1 h. Images were captured by the confocal microscope ZEISS LSM 880 Airyscan. λ_ex/em_ = 405/410–470 nm was used for DAPI, λ_ex/em_ = 488/500–542 nm was used for DiOC6(3), and λ_ex/em_ = 514/566–669 nm was used for Phalloidin-iFluor 555. Only the co-cultures with exogenous GFs on day 14 were further analyzed, as the other groups rarely formed rOCs. An example of the analysis is shown in Figure S4.

### 4.15. Statistical Analysis

In the case of rOBs characterization, six parallel wells for each of the six rats for the given isolation method were plotted in a box plot. In the case of osteogenic markers expression, three parallel samples for each of the six rats were analyzed. In the case of MC3T3-E1, six parallel wells were plotted. In the case of the co-cultures, five parallel wells for each of the four rats for the explant culture method or six rats for the explant culture with enzymatic pre-treatment method were plotted. Statistical analyses were performed in GraphPad Prism 9 using a Kruskal–Wallis test with a Dunn’s multiple comparison test. In the case of proliferation, metabolic activity, osteogenic marker expression, the number of rOCs, the area of rOCs, and the number of nuclei per rOC, the Mann-Whitney test was used. The statistical difference is marked as a link above the groups. * *p* < 0.05, ** *p* < 0.01, *** *p* < 0.001, **** *p* < 0.0001.

## Data Availability

Not applicable.

## Supplementary Materials

The following supporting information can be downloaded at: https://www.mdpi.com/article/10.3390/ijms23147875/s1.
