# Supplementary figures and images for: The Effect of Osteoblast Isolation Methods from Adult Rats on Osteoclastogenesis in Co-Cultures
Source: Int J Mol Sci. 2022 Jul 17;23(14):7875. doi: 10.3390/ijms23147875 (PMC9318333; doi:10.3390/ijms23147875)

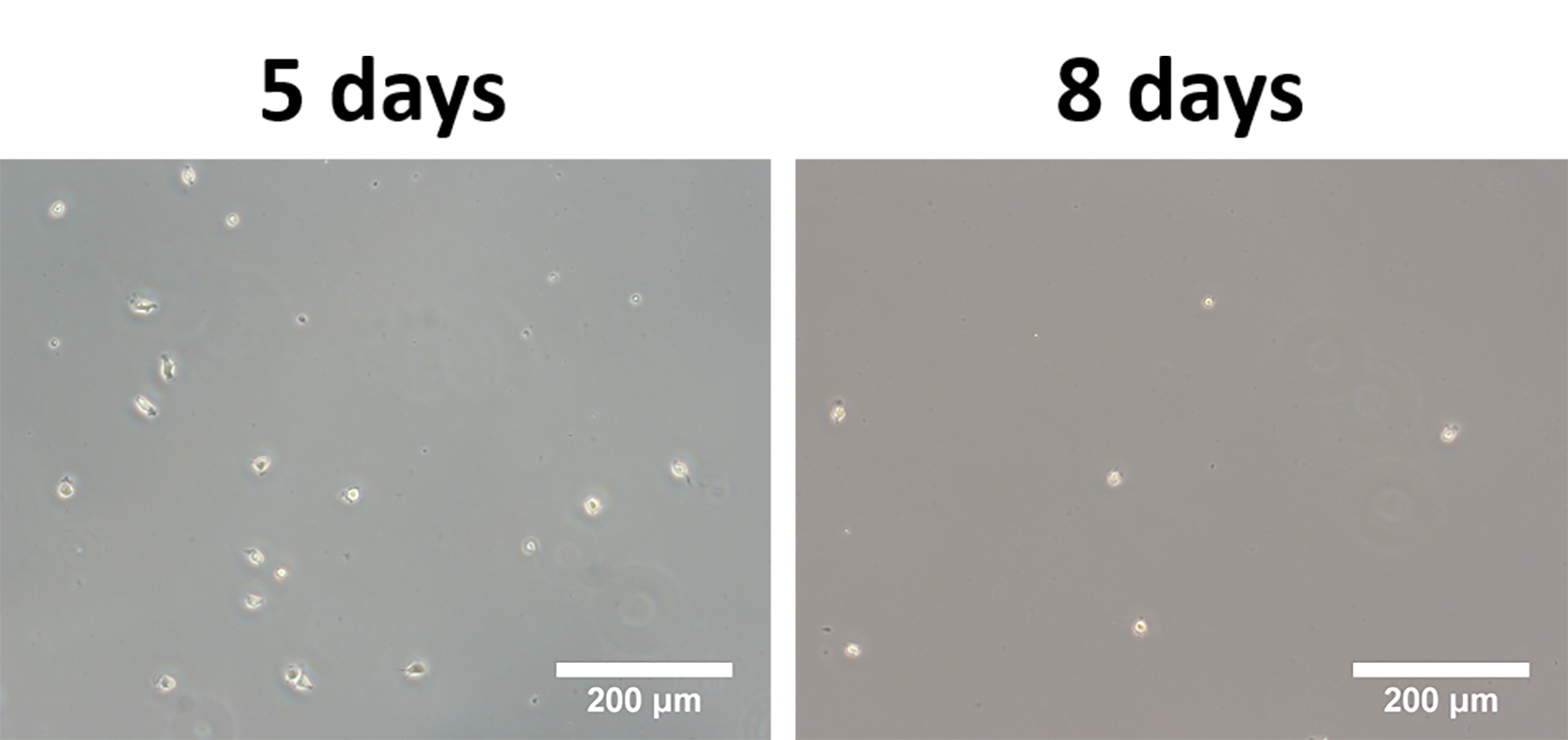

Supplement: Supplementary file 1 [file ijms-23-07875-s001.zip › Figure S1.tif]

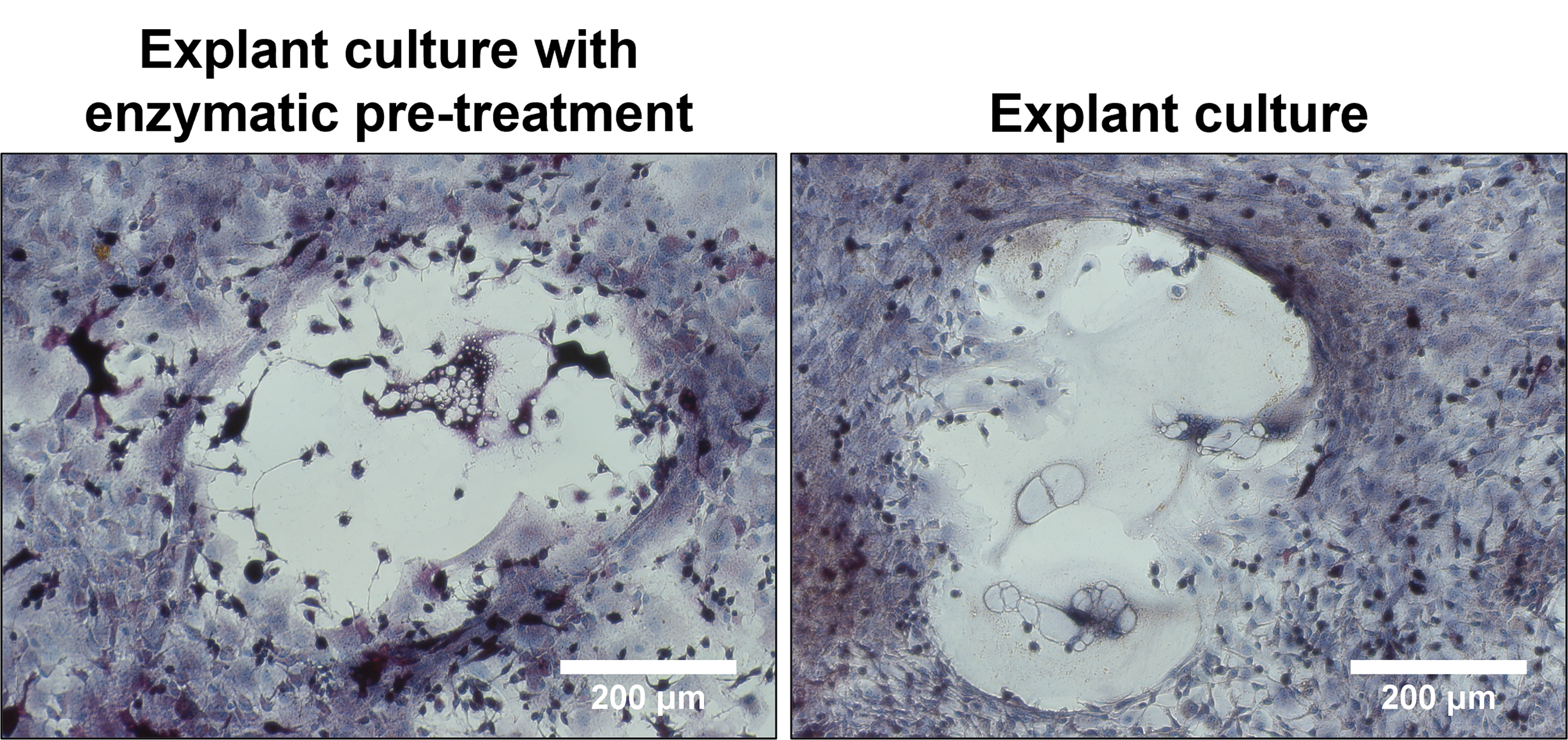

Supplement: Supplementary file 1 [file ijms-23-07875-s001.zip › Figure S2.tif]

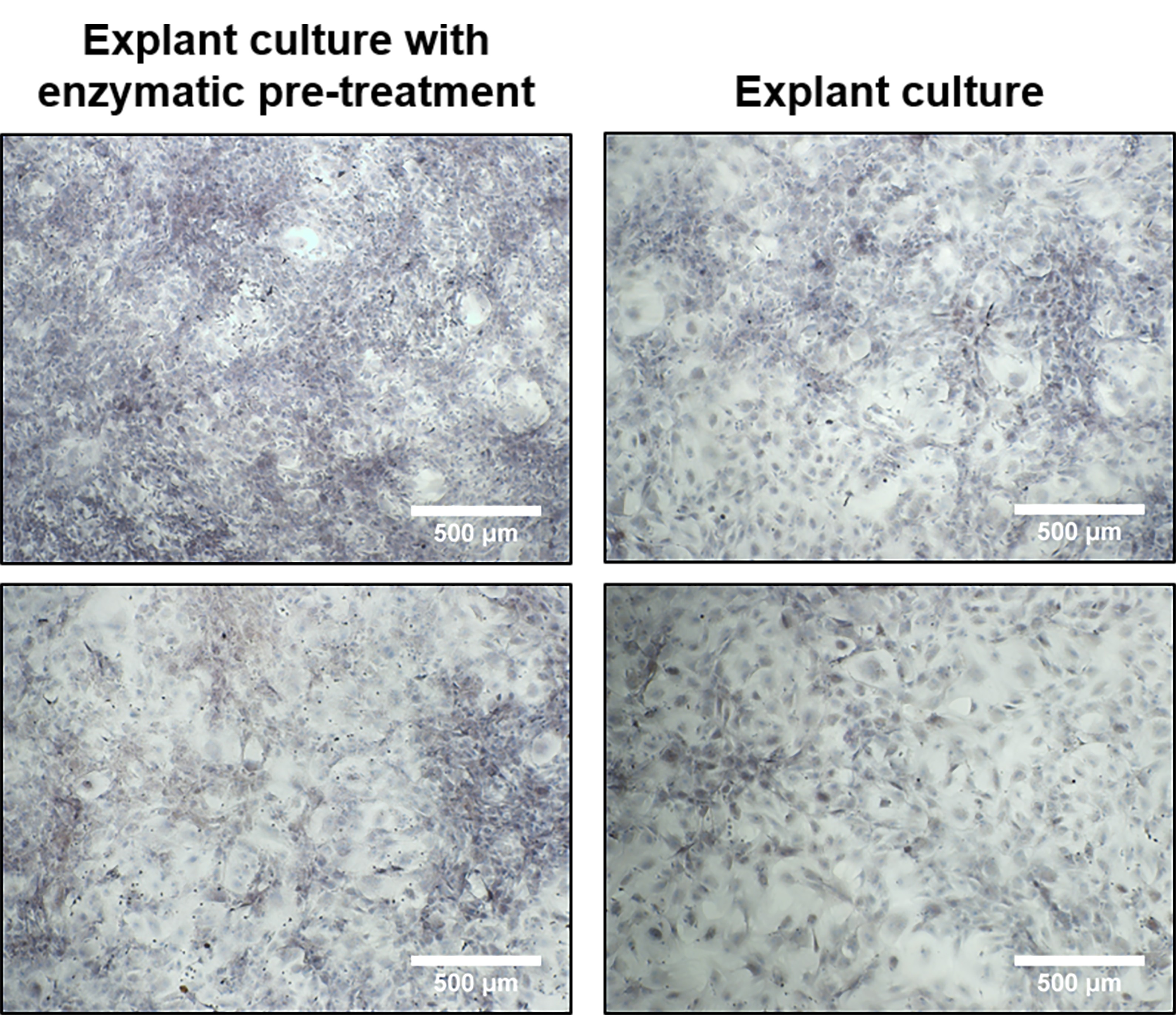

Supplement: Supplementary file 1 [file ijms-23-07875-s001.zip › Figure S3.tif]
